# Extracellular matrix promotes clathrin-dependent endocytosis of prolactin and STAT5 activation in differentiating mammary epithelial cells

**DOI:** 10.1038/s41598-017-04783-6

**Published:** 2017-07-04

**Authors:** Rebecca E. Bridgewater, Charles H. Streuli, Patrick T. Caswell

**Affiliations:** Wellcome Trust Centre for Cell-Matrix Research, Division of Cell Matrix Biology and Regenerative Medicine, School of Biological Sciences, Faculty of Biology Medicine and Health, University of Manchester, Manchester Academic Health Science Centre, Manchester, UK

## Abstract

The hormone prolactin promotes lactational differentiation of mammary epithelial cells (MECs) via its cognate receptor and the downstream JAK2-STAT5a signalling pathway. In turn this regulates transcription of milk protein genes. Prolactin signalling depends on a cross-talk with basement membrane extracellular matrix (ECM) via β1 integrins which activate both ILK and Rac1 and are required for STAT5a activation and lactational differentiation. Endocytosis is an important regulator of signalling. It can both enhance and suppress cytokine signalling, although the role of endocytosis for prolactin signalling is not known. Here we show that clathrin-mediated endocytosis is required for ECM-dependent STAT5 activation. In the presence of ECM, prolactin is internalised via a clathrin-dependent, but caveolin-independent, route. This occurs independently from JAK2 and Rac signalling, but is required for full phosphorylation and activation of STAT5. Prolactin is internalised into early endosomes, where the master early endosome regulator Rab5b promotes STAT5 phosphorylation. These data reveal a novel role for ECM-driven endocytosis in the positive regulation of cytokine signalling.

## Introduction

Cells within the tissues of multicellular organisms respond to positional signals from the extracellular matrix (ECM) and cell-cell adhesions, and to temporal signals from hormones, cytokines and growth factors^1^. In breast, joint signalling between extracellular matrix (ECM) and cytokines is critical for lactational differentiation. Whilst the mechanisms controlling signal integration are still being determined, an emerging theme is the involvement of endocytic trafficking pathways^2^.

Alveoli, which make milk proteins, are comprised of a layer of polarised mammary epithelial cells (MECs) surrounded by myoepithelial cells, and subtended by a laminin-rich basement membrane (LrBM). The engagement of LrBM ECM is crucial for MECs to respond to the lactation-inducing cytokine prolactin (Prl)^3^, however the mechanisms linking ECM and Prl signalling are not fully understood.

Prl binds to its cognate type I cytokine receptor (PrlR) at the plasma membrane (PM) of MECs to activate Janus kinase 2 (JAK2), resulting in phosphorylation of tyrosine residues on PrlR and recruitment of signal transducer and activator of transcription 5a (STAT5a)^4^. STAT5a then becomes phosphorylated by JAK2 on tyrosine 694 to induce dimerisation and nuclear translocation^5^. There, the STAT5a dimer causes transcription of milk protein genes, such as that encoding β-casein. Despite this detailed understanding to the molecular signalling pathway, the mechanism through which class I cytokine receptors are activated by ligand (dimerization versus conformational change), and the localisation of downstream signalling within the cell (plasma membrane versus endosomal platforms), remain enigmatic^6, 7^.

Endocytosis, mediated by clathrin-dependent and -independent mechanisms, has an important role in regulating receptor-mediated signalling both positively and negatively^8^. Endocytic trafficking can enhance signalling downstream of cytokine receptors and is implicated in the activation and nuclear translocation of STATs^9–14^. For example, mutations which retain granulocyte colony stimulating factor receptor (G-CSFR) in early endosomes of myeloid progenitor cells promote sustained STAT5 activity^15^. Furthermore, clathrin-mediated endocytosis (CME) is required for INFα receptor (a type II cytokine receptor) internalisation and STAT1/2 activation^16^. Signalling downstream of type I cytokine receptors is also facilitated by endocytosis. Caveolar endocytosis of growth hormone receptor (GHR, closely related to PrlR) is implicated in STAT-mediated transcription, and JAK2 associates with GHR following internalisation, suggesting that signalling continues on endosomes^17, 18^. Interleukin-5 (IL-5) receptor is internalised by CME and caveolar endocytosis, both of which are required for optimal downstream signalling via JAK2^19^. CME has been implicated in the endocytosis and degradation of PrlR^20^, and in breast cancer cells can enhance downstream signalling to ERK but not STATs^21^.

In MECs, Caveolin-1 (Cav1) associates with JAK2, and Cav1-null mice exhibit hyper-phosphorylation of STAT5 and precocious milk production^22^, suggesting a link between endocytic regulators and Prl signalling. However, the role of endocytosis in Prl signalling in MECs has not been investigated.

MECs interact with basement membrane ECM through β1-integrins, which are critical for Prl-stimulated PrlR-JAK2-STAT5 signalling^23^. Primary MECs isolated from β1-integrin conditional knockout mice do not synthesise β-casein, and β1-integrin-ILK-Rac signalling blocks the activation of SHP2, a phosphatase that inhibits JAK2^24–27^. Integrins are cargoes of endocytic trafficking pathways, but can also regulate endocytosis^28^. For example β1-ILK signalling promotes dynamin-dependent endocytosis of apical proteins from the basolateral membrane, thereby establishing polarity^29^. Similarly, in keratinocytes lacking β1 integrin or ILK, caveolae are absent from the PM due to a defect in microtubule targeting and stability^30^.

In this study we examine the hypothesis that LrBM impacts upon Prl signalling by controlling endocytosis and downstream signalling to STAT5. We show that in the presence of LrBM, Prl promotes STAT5 phosphorylation in a clathrin-dependent, caveolin-independent manner that requires Rab5b.

## Results

### Clathrin-mediated endocytosis is required for ECM-dependent Prl signalling

**Figure 1:**
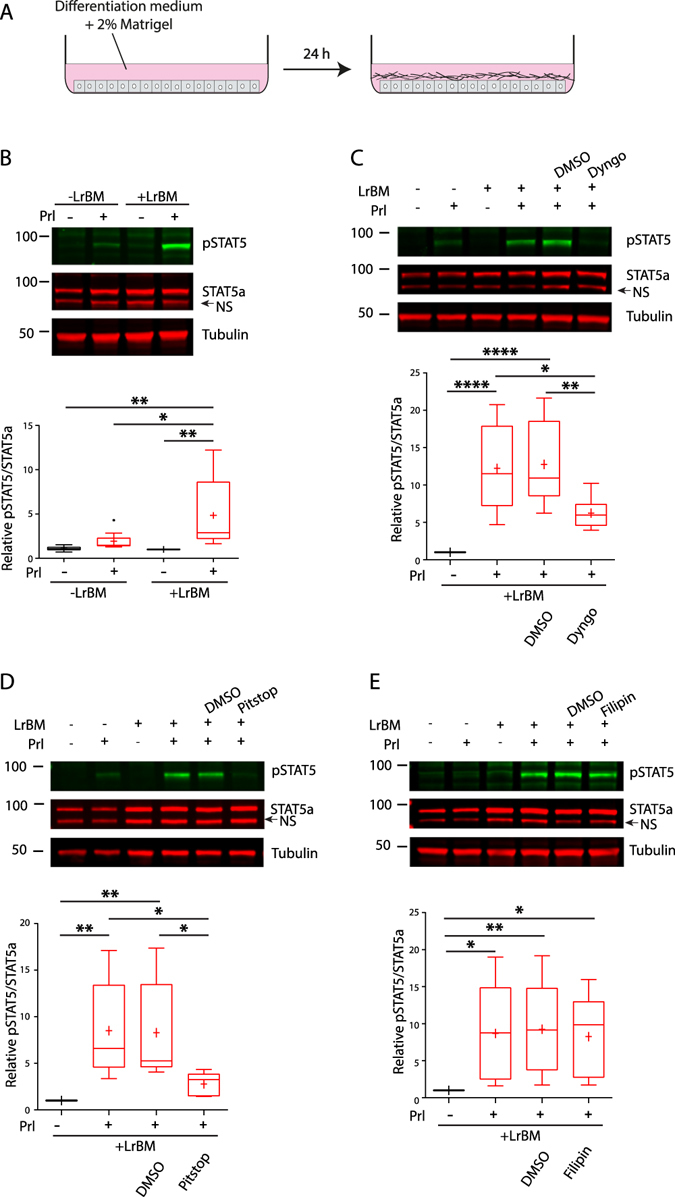
Inhibition of endocytosis suppresses STAT5 activation in MECs. (**A**) Schematic of 2D lactational differentiation model. (**B**) Eph4 cells were seeded onto plastic and LrBM added to the differentiation medium as appropriate. After 24 hours, cells were stimulated with Prl (3  μg/ml) as indicated for 15 mins before lysis. Samples were analysed by SDS-PAGE/western blotting with phospho-Y^694^ STAT5, total STAT5a or tubulin specific antibodies, and quantification of Odyssey scanned fluorescent images performed using ImageJ.(**C**–**E**): Eph4 cells as in (**A**) were treated with Dyngo4 (60 µM), Pitstop II (18 µM), Filipin III (8 µM) or DMSO as indicated prior to Prl stimulation, and analysed as in (**A**). Western blots are representative of, and graphs show normalised data from at least 3 independent experiments. *p < 0.05, **p.0.01 ****p.0.0001, ns = not significant.

LrBM ECM is required for Prl/STAT5a signalling in lactational differentiation. Eph4 cells, a MEC cell line derived from the mammary epithelium of pregnant mice, express β-casein when cultured in 3D LrBM extract^27^, and this is the only cell line model which robustly responds to ECM engagement and Prl in the way that primary cells do. When cultured on plastic dishes, they show little basal STAT5 phosphorylation, and only a modest increase upon stimulation with Prl (Fig. 1A,B). Culturing primary MECs in 2D with LrBM ECM overlay is sufficient to induce the expression of β-casein in response to Prl stimulation^23^. In Eph4 cells, ECM overlay promotes Prl-driven STAT5 phosphorylation (Fig. 1A,B), indicating that these cells can respond to lactational differentiation cues in 2D culture. In Eph4 cells and primary MECs, Prl stimulates STAT5 but not ERK phosphorylation (Supplementary Figure 1A,B), in contrast to findings in cancer cells^21^.

To investigate the role of endocytosis in Prl/ECM signalling through STAT5, cells were treated with inhibitors of clathrin-dependent and -independent endocytosis pathways. In the presence of Dyngo4, an inhibitor of dynamin (a large GTPase required for both clathrin- and caveolin-dependent endocytosis), there were only low levels of STAT5 phosphorylation in response to Prl and ECM (Fig. 1C). Endocytosis is therefore key to the correct activation of signals downstream of Prl.

Inhibition of CME with Pitstop-2 also prevented Prl/ECM-induced STAT5 activation to levels similar to those seen with only Prl, without ECM (Fig. 1D). Equivalent results were obtained with an alternative CME inhibitor monodansylcadaverine (MDC, not shown). Inhibition of CME or Dynamin were also observed to suppress STAT5 activation in primary MECs (Supplementary Figure 1B). By contrast, cholesterol sequestration with Filipin, which suppresses caveolar endocytosis and macropinocytosis, had no effect on Prl signalling (Fig. 1E).

**Figure 2:**
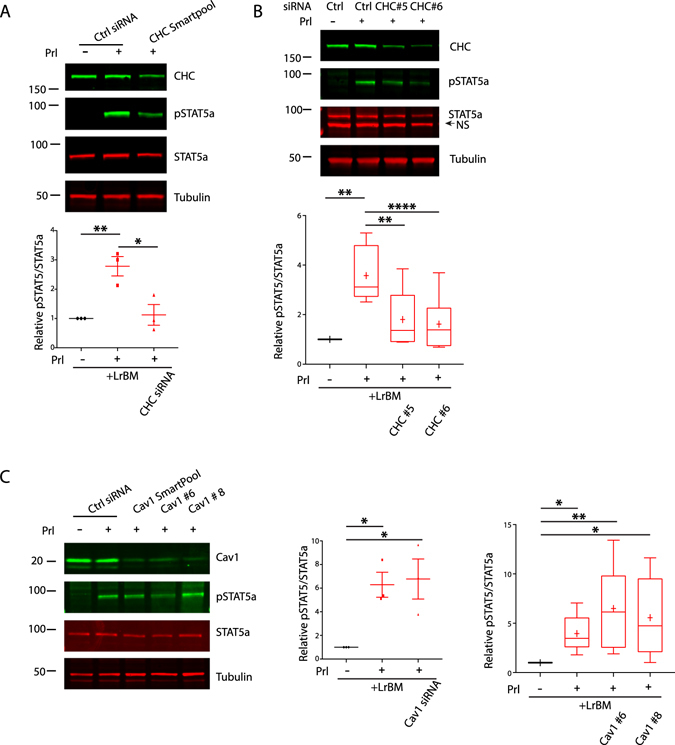
Clathrin promotes Prolactin-induced STAT5 activation in LrBM-engaged MECs. (**A**,**B**) Eph4 cells transfected with CHC-specific SMARTpool (**A**) or individual oligos (**B**) were seeded onto plastic and LrBM added to the differentiation medium as appropriate. After 24 hours, cells were stimulated with Prl (3 μg/ml) as indicated for 15 mins before lysis. Samples were analysed by SDS-PAGE/western blotting with phospho-Y^694^ STAT5, total STAT5a or tubulin specific antibodies, and quantification of Odyssey scanned fluorescent images performed using ImageJ. (**C**) Eph4 cells transfected with Caveolin-1-specific SMARTpool or individual oligos were treated and analysed as in (**A**). Western blots are representative of, and graphs show normalised data from, at least 3 independent experiments. *p.0.05, **p < 0.01, ns = not significant.

To confirm the requirement for clathrin-dependent endocytosis in Prl signalling, a small interfering RNA (siRNA) was used to deplete clathrin heavy chain (CHC). Efficient CHC knockdown, using a SmartPool designed to reduce off-target effects, significantly reduced the phosphorylation of STAT5 in response to Prl stimulation (Fig. 2A). Individual oligonucleotides (oligo#5 and #6) that efficiently deplete CHC confirmed this (Fig. 2B; note that re-expression rescue is not possible in this differentiation model). By contrast caveolin-1 knockdown with either SMARTpool or either of two individual siRNA oligos had no influence on Prl/ECM-induced STAT5 phosphorylation (Fig. 2C). Because clathrin is not only involved in endocytosis^31^, we also targeted the major clathrin adaptor AP2, depleting the AP2M1 subunit by RNAi. AP2M1 knockdown significantly reduced STAT5 phosphorylation in response to LrBM and Prl (Supplementary Figure 1C), confirming that clathrin-dependent endocytosis plays an important role in STAT5 phosphorylation and activation.

These results show that, clathrin- but not caveolar-, mediated endocytosis is required for Prl/ECM signalling and STAT5 phosphorylation.

### Prolactin is internalised in a clathrin-dependent manner

**Figure 3:**
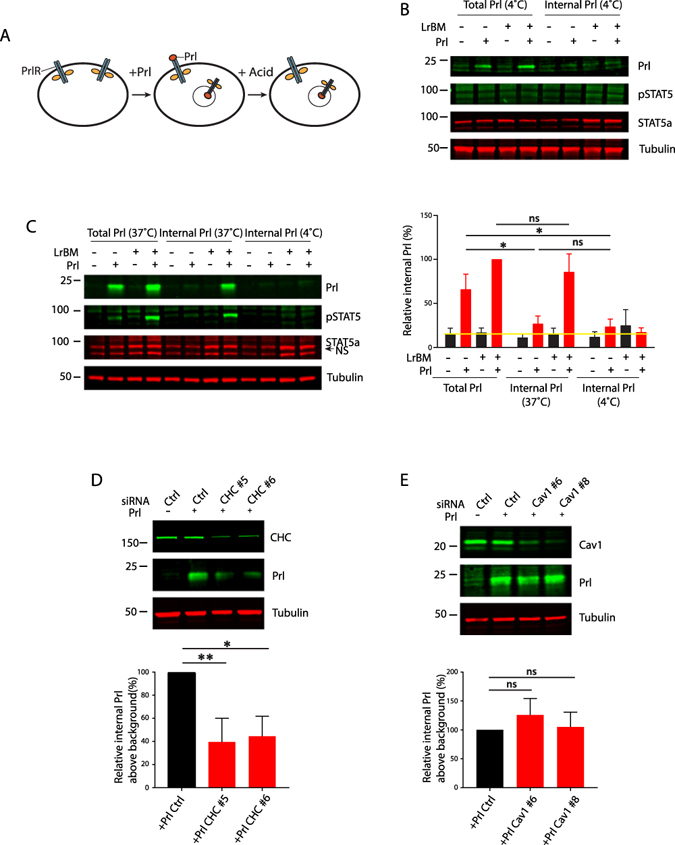
Prolactin is internalised via CME in LrBM-engaged MECs. (**A**) Schematic of Prl internalisation assay. (**B**) Eph4 cells seeded onto plastic were overlaid with LrBM as appropriate and labelled with Prl (3  μg/ml) at 4 °C for 15 mins. Cells were either washed with PBS to reveal total bound Prl (Total), or acid washed to reveal internalised Prl (Internal), and samples analysed by SDS-PAGE and western blotting with Prl, pSTAT5, STAT5 and tubulin specific antibodies. (**C**) Eph4 cells were labelled with Prl (3 μg/ml) for 15 mins at 37 °C or 4 °C as indicated, and total and internal pools analysed as in (**B**). Quantification of Odyssey scanned fluorescent images performed using ImageJ. Yellow line indicates background signal in the absence of exogenous Prl. (**D**,**E**) Prl internalisation was analysed in CHC and Caveolin-1 knockdown cells (2 oligos each), background signal in the absence of exogenous Prl was subtracted to give relative internal Prl above background. Western blots are representative of, and graphs show normalised data from, at least 3 independent experiments. *p < 0.01, **p.0.001, ns = not significant.

Because CME is implicated in the endocytosis of cargoes that include cytokine receptors, we determined whether Prl was internalised during lactational differentiation. We monitored Prl internalisation as a surrogate for PrlR, because of the lack of reagents available to detect mouse PrlR. Cells cultured in the presence or absence of LrBM overlay were stimulated with Prl, and acid washed to remove surface Prl, leaving only internalised Prl (Fig. 3A). Prl labelled the cell surface receptor efficiently at 4 °C, but no internalisation occurred and surface Prl could be efficiently removed (Fig. 3B). By labelling at 37 °C, we observed a broadly similar level of recruitment of Prl to its cell-surface receptor (Fig. 3C; -LrBM + Prl vs. + LrBM + Prl). However Prl was only internalised in cells cultured with LrBM at physiological temperature, demonstrating that ECM is required for Prl-endocytosis.

Using siRNA-mediated knockdown, we investigated the route of endocytic entry of Prl. In accordance with the requirement for CME in STAT5 activation, knockdown of CHC using two individual oligos significantly reduced Prl internalisation (Fig. 3D). Because CHC knockdown did not completely abrogate Prl internalisation, we investigated alternative endocytic routes. Caveolin-1 knockdown had no influence on Prl internalisation (Fig. 3E), suggesting that CME is a major route of Prl endocytosis which controls Prl signalling to STAT5, although other clathrin-independent routes could play a more minor role. Importantly, knockdown of CHC or Caveolin-1 did not influence the recruitment of Prl to the cell surface (Supplementary Figure 1D,E).

These results show that LrBM ECM induces Prl internalisation via a clathrin-dependent, but caveolin-independent route.

### Active JAK2 and Rac1 are not required for Prl internalisation

**Figure 4:**
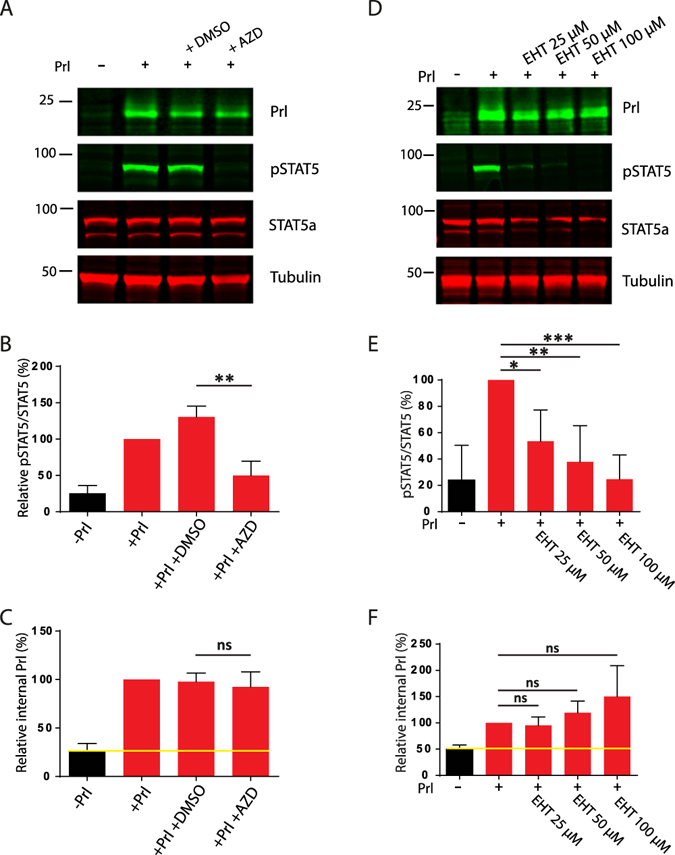
Inhibition of Rac or JAK2 prevents STAT5 phosphorylation without influencing prolactin endocytosis. Eph4 cells were seeded onto plastic and LrBM added to the differentiation medium as appropriate. After 24 hours, cells were treated with inhibitors (JAK2 inhibitor (AZD-1480, 10 µM; (**A**–**C**) or Rac inhibitor (EHT 1864, 25–100 µM; D-E)) for 15 mins and then stimulated with Prl (3 μg/ml) as indicated for 15 mins before cells were acid washed to remove surface Prl and lysed. Samples were analysed by SDS-PAGE/western blotting with phospho-Y^694^ STAT5, total STAT5a, Prl or tubulin specific antibodies, and quantification of relative STAT5 phosphorylation (**B**,**E**) and Prl internalisation (**C**,**F**) from Odyssey scanned fluorescent images performed using ImageJ. Yellow line indicates background signal in the absence of exogenous Prl. Western blots are representative of, and graphs show normalised data from, at least 3 independent experiments. *p < 0.01; **p.0.001, ***p.0.0001, ns = not significant.

Since JAK2 activation has been implicated in PrlR internalisation in cancer cells^32^, we elucidated other mechanisms that could be involved with Prl internalisation. However, inhibition of JAK2 with its specific inhibitor AZD-1480 had no effect on Prl internalisation in cells cultured with ECM overlay, despite a 60% reduction in the phosphorylation of STAT5 on Y694 (Fig. 4A–C).

We previously showed that an ILK-Pix-Rac signalling axis is of critical importance to Prl-induced STAT5 activation and milk protein production. We therefore investigated the role of Rac in Prl internalisation and Prl signalling. Preventing Rac signalling with the drug EHT1864 inhibited Prl-induced STAT5 phosphorylation in ECM-engaged MECs (Fig. 4D,E). However, Rac inhibition had no influence on Prl internalisation (Fig. 4D,F). Thus whilst ECM is critical for Prl internalisation, signalling via Rac1 is not required for Prl endocytosis.

These results show that ECM promotes endocytosis of Prl, and at the same time elicits β1-integrin signalling via Rac (to suppress the JAK2 inactivator SHP2). Together these drive STAT5 phosphorylation and activation.

### Internalisation of prolactin into early endosomes promotes STAT5 phosphorylation

**Figure 5:**
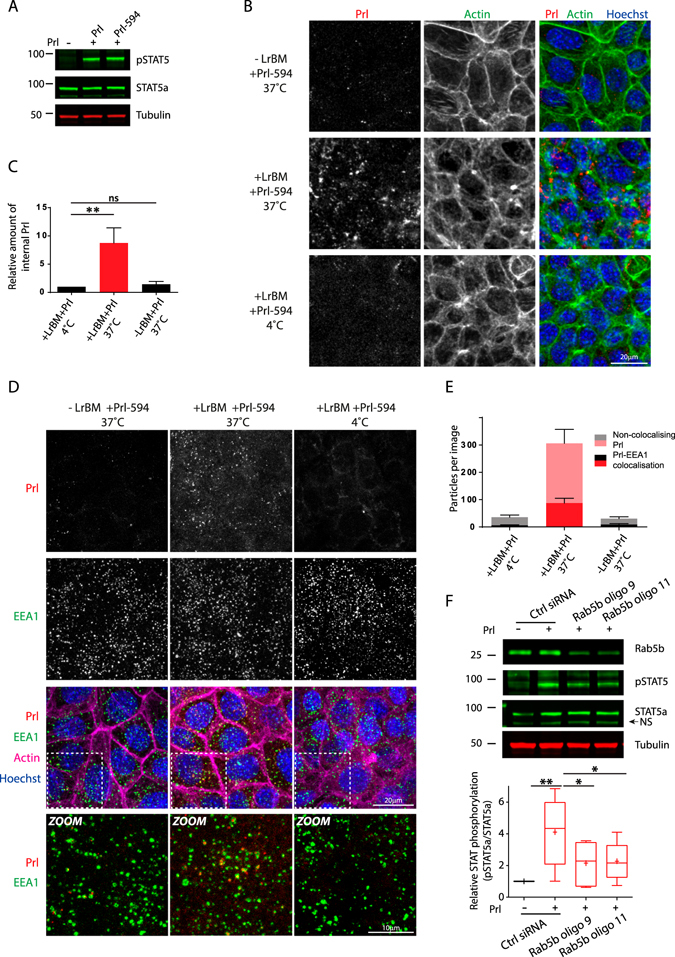
Prolactin internalisation into early endosomes is required for Prl-signalling in differentiating MECs. (**A**) Eph4 cells were seeded onto plastic and LrBM added to the differentiation medium as appropriate. After 24 hours, cells were stimulated with unlabelled or labelled Prl (3 μg/ml) as indicated for 15 mins before lysis. Samples were analysed by SDS-PAGE/western blotting with phospho-Y^694^ STAT5, total STAT5a or tubulin specific antibodies. (**B**–**E**) Eph4 cells were seeded on glass coverslips and LrBM added to the differentiation medium as appropriate. After 24 hours, cells were stimulated with labelled Prl (3 μg/ml) as indicated for 15 mins before fixation and staining for actin and nuclei (**B**) or EEA1 and nuclei (**D**). Confocal stacks were captured and representative maximum intensity projections are shown. Internalised Prl particles (**C**) and internalised particles co-localised with EEA1 (**E**) were analysed using ImageJ. F: Rab5b knockdown cells were treated and STAT5 phosphorylation determined as in (**A**) and quantification of Odyssey scanned fluorescent images performed using ImageJ. Western blots and images are representative of, and graphs show normalised data from, at least 3 independent experiments. *p < 0.01, **p.0.001, ns = not significant.

To image internalised Prl, we fluorescently labelled Prl with Alexafluor-594. We confirmed that labelled Prl (i.e. Prl-594) could induce STAT5 phosphorylation to the same extent as unlabelled-Prl (Fig. 5A). MECs cultured with or without ECM were treated with Prl-594 for 15 minutes, then acid-washed to reveal intracellular Prl accumulation. In the presence of ECM at 37 °C, Prl-594 was found in small, intracellular punctae throughout the cytoplasm, consistent with a vesicular/endosomal localisation (Fig. 5B). Quantification revealed an 8-fold increase in internal Prl punctae compared to cells labelled and chased at 4 °C to suppress internalisation (Fig. 5C). In the absence of ECM there was no significant increase of Prl endocytosis compared to the non-internalised (4 °C) control (Fig. 5C). These data show that ECM promotes internalisation of Prl into vesicles/endosomes.

Because CME derived vesicles transit to early endosomes before for sorting for degradation or recycling within 5–15minutes^8^ (consistent with the peak in STAT5 phosphorylation and activation), we investigated the localisation of internalised Prl with respect to the early endosome marker, early endosome antigen 1 (EEA1). Cells showed little Prl endocytosis in the absence of ECM or at 4 °C, and little overlap between any internal Prl structures and EEA1. However, ECM promoted significant colocalisation of internalised Prl with EEA1-postive early endosomes at physiological temperature (Fig. 5D,E).

Early endosomes are platforms for sorting of cargos via recycling or degradative routes, and they also function to regulate signalling downstream of several receptor classes^33^. The Rab5 family of small GTPases are master regulators of early endosome biogenesis and function^34^, and the major isoform expressed in the lactating mammary gland is Rab5b^35^. To further investigate the function of Prl endocytosis in downstream signalling we used siRNA to knock down Rab5b. Using two separate oligos we found that there was a significant and consistent reduction in STAT5 phosphorylation (Fig. 5F). These data indicate that in the presence of LrBM ECM, Rab5b-mediated internalisation of Prl into early endosomes is required for STAT5 phosphorylation.

Together these results show that endocytosis of Prl signalling complexes is required for the complete phosphorylation and activation of STAT5, and may underpin the programme of lactational differentiation in MECs.

## Discussion

We have discovered that clathrin-, but not caveolar-, mediated endocytosis is key to the efficient activation of STAT5 downstream of Prl in MECs. LrBM ECM promotes internalisation of Prl into early endosomes, and Rab5b is required for STAT5 activation. ECM therefore provides signals to permit STAT5 activation by controlling endocytosis of Prl.

Endosomes are key signalling platforms for several pathways, assembling cascades via adaptor molecules and scaffolds, which facilitate signal amplification^8^. In Drosophila, clathrin-mediated endocytosis is required for efficient JAK-STAT signalling in border cells^11^, and in mammalian systems endosomal signalling is important for sustaining STAT3 activity^13, 14^. We show that Prl transits through early endosomes at the same time as STAT5 phosphorylation is intensified. Thus, as is the case for other signalling cascades, endosomes act as key regulators of signal propagation in the Prl/ECM-mediated STAT5 pathway in MECs.

GHR is closely related to PrlR, and caveolar endocytosis has been implicated in its downstream signalling^17, 18^. However caveolin knockout mice exhibit hyper-phosphorylation of STAT5a, suggesting that there could be important differences in the mechanism controlling downstream signalling^22^. We have discovered that clathrin-dependent endocytosis is required for signalling downstream of a Type I cytokine receptor, PrlR. This has previously only been seen for Type II cytokine receptors^16^. In our experiments, knockdown of CHC (or AP2) does not completely abrogate STAT5 phosphorylation, suggesting that while clathrin-dependent endocytosis is of important, clathrin-independent pathways could also play a role.

Interestingly in cancer cell lines, PrlR signalling and internalisation is not dependent upon ECM, and CME is involved in down-regulation of PrlR^20, 32, 36^. In this cancer context, JAK2 activation is implicated in the control of internalisation, but JAK2-STAT5 signalling does not require endocytosis. Moreover, whilst Prl elicits JAK2-STAT5 signalling and ERK signalling in cancer cells, normal MECs do not produce an ERK response, suggesting that important differences in the regulatory mechanisms controlling PrlR signalling exist in different contexts. Our results therefore reveal distinctions between Prl internalisation and signalling in tumorigenic versus non-tumorigenic cells, which could be exploited for therapeutic benefit.

Integrins elicit a plethora of signalling responses upon engagement of their ECM ligands, and we and others have previously shown that they are involved in a wide variety of signalling responses initiated by growth factors^3, 37, 38^. Integrin signalling can also control endocytosis and signalling of PDGF and BMP receptors^39, 40^. The Rac signalling axis is not required for Prl endocytosis, and likely acts via phosphatases that control JAK2^25^, suggesting that ECM impacts on Prl endocytosis via an alternate route (perhaps through β1-integrins or other LrBM receptors). For example a Rac1-independent, β1-ILK-microtubule pathway controls tight junction protein internalisation in polarising MECs^29^, and in keratinocytes a similar pathway controls plasma membrane distribution of caveolae^30^. Alternate integrin-dependent signalling pathways may therefore regulate Prl endocytosis downstream of LrBM ECM.

In summary, this study demonstrates that basement membrane ECM influences Prl internalisation through a clathrin-mediated endocytic route. Delivery to an early endosomal platform promotes downstream signalling to STAT5, the master transcriptional regulator of lactational differentiation. We deliver critical new insight into the ECM-mediated regulation of hormonal signalling in the mammary gland, and reveal the Prl/PrlR/JAK2/STAT5 signalling axis as a beneficiary of endosomal signalling.

## Conclusions

Prl and ECM are both required for tissue-specific gene expression in MECs, and we reveal that their signalling pathways communicate via endocytosis and early endosomes.

- Endocytosis is central for Prl/ECM signalling.
- Clathrin- but not caveolar-, mediated endocytosis is required for Prl/ECM signalling.
- Internalisation of prolactin into early endosomes via a Rab5b pathway is required for STAT5 phosphorylation.

## Materials and Methods

### Cell culture and siRNA knockdown

Low passage EpH4 cells^41^ were cultured in Dulbecco’s Modified Eagle Medium (DMEM) supplemented with 10% (v/v) foetal calf serum (FCS), 5 μg/ml insulin and antibiotics. Two rounds of knockdown were performed to achieve protein suppression: 100 pmol siRNA (Dharmacon ON-TARGETplus SmartPools and individual oligos: Control pool #001810-10, Control single oligo #001810-01-05, Clathrin heavy chain SMARTpool #063956-00, Oligo#5 063956-05, Oligo#6 063956-06; Caveolin-1 SMARTpool 058415-00, Oligo#6 058415-06, Oligo#8 058415-08; Rab5b Oligo#9 040856-09, Oligo#11 040856-11; AP2M1 RNAi 5′- AAGUGGAUGCCUUUCGGGUCA^42^) was used to transfect a single well of a six well plate of EpH4 cells and cells were reseeded 24 hours later for a second round of knockdown at 48 hours, and experiments were performed and/or cells harvested at 72 hours. Primary MECs were isolated and cultured as described^25^.

### STAT5 phosphorylation

For differentiation assays, EpH4 cells were cultured in DMEM-F12 supplemented with 5 µg/ml insulin, 1  μg/ml hydrocortisone and antibiotics overnight with 2% Matrigel (LrBM, BD Biosciences) added to the culture medium (LrBM overlay) as required. Cells were stimulated with 3 µg/ml sterile-filtered sheep pituitary Prl (Sigma #L6520; 150 nM final Prl concentration) for 15 minutes. EpH4 cells were treated with inhibitors (Pitstop-2 [15 µM, Abcam]; Dyngo-4a [60 µM, Abcam]; Filipin III [8 µM, Sigma-Aldrich]; AZD-1480 [10 µM, Santa Cruz Biotechnology]; EHT 1864 [25–100 µM, Tocris]) or an equal volume of DMSO (where appropriate) diluted in differentiation medium for 15 minutes at 37 °C before addition of Prl.

### Western blotting

Sample buffer (50 mM Tris-HCl pH 6.8, 10% (w/v) glycerol, 4% (w/v) SDS, 0.004% (w/v) bromophenol blue) with 10% (v/v) 2-mercaptoethanol (Sigma-Aldrich) was added to samples, which were then incubated at 95 °C for 10 minutes. The samples were run alongside Precision Plus Protein All Blue Standards (Bio-Rad Laboratories) on 4–12% SDS polyacrylamide gels (Life Technologies) at 100 V. The gels were transferred to nitrocellulose membrane at 30 V for 75 minutes. Membranes were incubated with a casein-based blocking buffer (Sigma-Aldrich) or 5% BSA in Tris-buffered saline with 0.1% Tween-20 (TBST; for phospho-specific antibodies) for 45 minutes. The membranes were incubated with primary antibodies or Alexa Fluor 680–conjugated streptavidin (1:2000; Invitrogen) in blocking buffer overnight at 4 °C.

Proteins levels were analysed by western blotting using the following primary antibodies: pSTAT5 (Y694, #9351, CST); pERK1/2 (#4370, CST), ERK1/2 (#9102, CST), ERK2 (sc-154) STAT5a (sc-1081) and Rab5b (sc-598, Santa Cruz Biotechnology); Prl (ab960, Millipore); Caveolin 1 (#610060, BD Biosciences); Clathrin heavy chain (ab172958), α-tubulin (ab6160, Abcam) and AP2M1 (mouse anti-AP50 Clone 31, BD Transduction Labs). Secondary antibodies used were: anti-rabbit Alexa Fluor 680 (A-21109), anti-mouse Alexa Fluor 680 (A-21058), and anti-rat Alexa Fluor 680 (A-21096, Invitrogen); anti-mouse DyLight 800 (#5257, Cell Signaling); anti-rat Alexa Fluor 800 (STAR71D800GA, AbD Serotec). Fluorescent secondary antibodies were detected by Odyssey scanner (Li-Cor), and intensity of bands quantified using ImageJ.

### Alexa Fluor–labelling of Prl

10 μg/ml Prl (Sigma-Aldrich; dissolved in water) was dialysed into 0.1 M NaHCO_3_ and incubated with 1 mg Alexa Fluor 488 or 594 NHS Ester (Succinimidyl Ester; Life Technologies, in 100 μl DMSO) for 1 h at room temperature. The labelled Prl was then dialysed into PBS.

### Prl internalisation assay

EpH4 cells cultured with a BM overlay were stimulated with 3 μg/ml Prl or Alexa Fluor-labelled Prl for 15 minutes at 37 °C to allow internalisation or at 4 °C to prevent internalisation. The cells were washed three times for three minutes each with PBS (Total) or with PBS-HCl pH 4.0 to remove the surface Prl (Internal). The cells were then washed three times with PBS and lysed or fixed.

### Immunofluorescence

Cells were fixed in 4% paraformaldehyde (PFA) before permeabilisation and blocked with 1% BSA in PBS for 30 minutes. Primary antibody (EEA1 #3230 CST) was diluted PBS/1% BSA and detected with fluorophore-conjugated secondary antibodies, actin using fluorophore labelled Phalloidin (Invitrogen) and nuclei with DAPI. Z-stacks were obtained using the 64x objective of a Leica TCS SP5 inverted confocal microscope. Images were processed using ImageJ.

For quantification of internalisation, background was subtracted using a radius of 100 pixels, and the threshold set to 100 pixels to produce a binary (black and white) image. The number of particles was counted automatically using ImageJ, and normalised to the area selected. For quantification of co-localisation, the background was subtracted and threshold set as above, and an image of the overlap between the two binary images was produced using Image Calculator. The number of particles was counted automatically using ImageJ, and calculated as a percentage of the number of particles in the Prl image.

### Statistical analysis

Statistical analysis was performed using GraphPad Prism. Analysis of variance (ANOVA) and post-hoc analysis was used to assess significance of data. Data are presented in box and whisker plots (Tukey) or bar graphs as mean −/+ standard error of the mean (SEM).

### Data availability

The datasets generated during and/or analysed during the current study are available from the corresponding author on reasonable request.

## Electronic supplementary material

Supplementary Information
