# Supplementary material for: Extracellular matrix promotes clathrin-dependent endocytosis of prolactin and STAT5 activation in differentiating mammary epithelial cells
Source: Sci Rep. 2017 Jul 4;7:4572. doi: 10.1038/s41598-017-04783-6 (PMC5496899; doi:10.1038/s41598-017-04783-6)
Supplement: Supplementary file 1 — Supplementary Information [file 41598_2017_4783_MOESM1_ESM.pdf]

Supplementary information:

Extracellular matrix promotes clathrin-dependent endocytosis of prolactin and STAT5 activation in differentiating mammary epithelial cells

Rebecca E. Bridgewater, Charles H. Streuli and Patrick T. Caswell*

Wellcome Trust Centre for Cell-Matrix Research,
Division of Cell Matrix Biology and Regenerative Medicine,
School of Biological Sciences,
Faculty of Biology Medicine and Health,
University of Manchester,
Manchester Academic Health Science Centre,
Manchester, U.K.

*Corresponding author: patrick.caswell@manchester.ac.uk

Running title: ECM activates STAT5 via Prl endocytosis

Supplementary Figure 1, Bridgewater et al. 2017

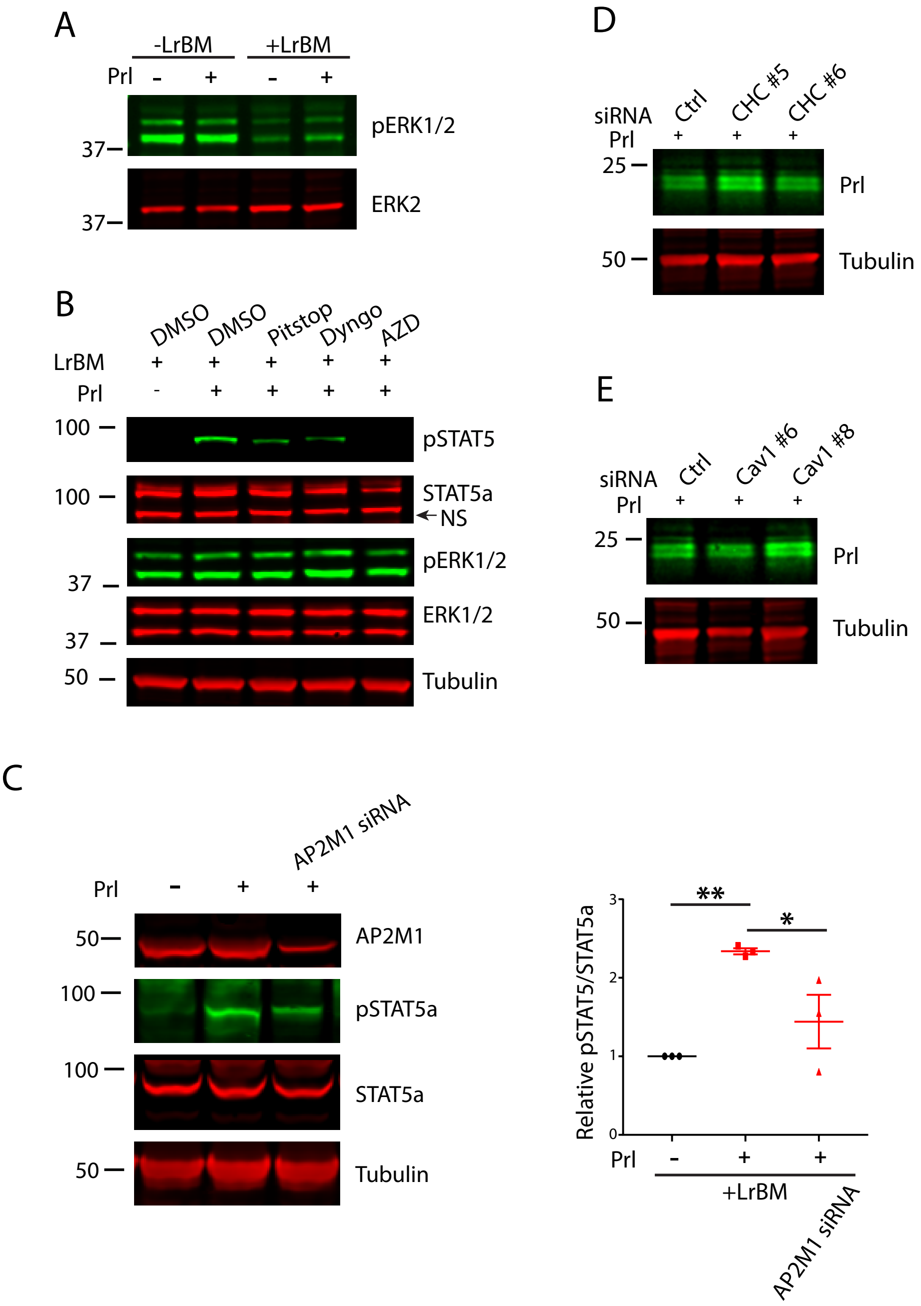

Supplementary Figure 1

A: Eph4 cells were seeded onto plastic and LrBM added to the differentiation medium as appropriate. After 24 hours, cells were stimulated with Prl (3μg/ml) as indicated for 15 mins before lysis. Samples were analysed by SDS-PAGE/western blotting with phospho-ERK1/2 or total ERK2 specific antibodies. B: Primary MECs were seeded onto plastic and LrBM added to the differentiation medium as appropriate. After 24 hours, cells were stimulated with Prl (3μg/ml) as indicated for 15 mins before lysis. Samples were analysed by SDS-PAGE/western blotting with phospho-Y694 STAT5, total STAT5a, phospho-ERK1/2, total-ERK1/2 or tubulin specific antibodies. C: Eph4 cells transfected with an AP2M1-specific RNAi oligonucleotide were seeded onto plastic and LrBM added to the differentiation medium. After 24 hours, cells were stimulated with Prl (3μg/ml) as indicated for 15 mins before lysis. Samples were analysed by SDS-PAGE/western blotting with phospho-Y694 STAT5, total STAT5a or tubulin specific antibodies, and quantification of Odyssey scanned fluorescent images performed using ImageJ. D, E: Prl internalisation was analysed as in Figure 3B, C in CHC and Caveolin-1 knockdown cells (2 oligos each).
